# Certifying Gender Equality in Research: Lessons Learnt From Athena SWAN and Total E-Quality Award Schemes

**DOI:** 10.3389/fsoc.2021.784446

**Published:** 2021-11-16

**Authors:** Charikleia Tzanakou, Kate Clayton-Hathway, Anne Laure Humbert

**Affiliations:** Centre for Diversity Policy Research and Practice, Oxford Brookes Business School, Oxford Brookes University, Oxford, United Kingdom

**Keywords:** certification, award, gender equality, higher education, research, Europe, Athena SWAN

## Abstract

In the past 2 decades, many Certification and Award schemes (CAS) related to gender equality, diversity and inclusion have emerged in the higher education, research and industry sectors. According to a recent report, there are as many as 113 CAS which have been identified across Europe and beyond. These CAS aim at addressing inequalities in relation to the grounds of sex, gender, race, sexual orientation, and disability among others. The high number of CAS, and their continued growth, has taken place in parallel to the shift of policies and efforts from “fixing individuals” to “fixing the system.” In these schemes, gender equality is often understood as a structural, systemic challenge, with a recognition that advancing gender equality is complex and requires drivers and interventions at micro, meso and macro level. Studies focused on analysing and evaluating gender equality initiatives in higher education have been scarce, and often limited to specific schemes. This paper aims to fill this gap by providing a better understanding of the CAS landscape through comparing two of the main gender equality schemes used by research-performing organisations in Europe Athena SWAN (in the UK) and Total E-Quality Award (in Germany). Based on qualitative interviews with stakeholders across Europe and document analysis, this paper focuses on strengths, challenges faced by and the impact of these CAS. This comparative exercise highlights particular learning points that can inform potential reviews of existing schemes and/or the development of new schemes such as a Europe-wide scheme. The latter is the focus of a Horizon 2020 project entitled CASPER (Certification-Award Systems to Promote Gender Equality in Research), which aims at making recommendations to the European Commission as to the feasibility of a Europe-wide CAS for gender equality in research organisations.

## Introduction

Higher education and research organisations are increasingly undertaking gender equality efforts to address entrenched inequalities in the academic system. Those efforts are often translated in Gender Equality Plans (GEPs) which are perceived as significant mechanisms for organisational change and gender equality (EIGEECC, 2012; EIGEECC, 2016; Clavero and Galligan, 2021). A gender action plan is considered as a set of actions which aim to “conduct impact assessment/audits of procedures and practices to identify gender bias; implement innovative strategies to correct any bias; set targets and monitor progress *via* indicators” (ECC, 2012, p.13).

At European level, the European Commission has awarded an increasing amount of European funding (since the sixth Framework programme) on cross-national consortia aimed at providing resources to institutional teams to design and implement GEPs. These funding programmes - Coordination and Support Actions-are aimed at triggering structural and cultural change in Higher Education Institutions (HEIs) across Europe, knowledge exchange and the dissemination of good practices in the wider European higher education community. In addition, many research programmes have been funded to better understand implementation and evaluation of gender equality efforts such as CASPER (Certification Award Systems to Promote Gender Equality in Research), which aims at exploring the feasibility of a Europe-wide scheme on gender equality through mapping and assessing existing Certification and Award schemes (CAS), on which this paper draws.

Certification also often requires a gender action plan, and this is typically a central feature of any assessment mechanism within each scheme. Because CAS predominantly operate at national level (with the exception of some that have been transferred and tailored to other national contexts), there is a plurality of formats, understandings and priorities that co-exist. Increasingly, however, existing schemes highlight gender equality as a structural issue in research organisations hence mirroring the focus of the EC. While they initially focused on careers and other HR-related gender equality issues, there is a growing recognition of other topics and/or a questioning of the concept of excellence in research and innovation. The growth and endorsement of these schemes by various national and European organisations also reflect the shift of policies and efforts from “fixing individuals” into “fixing the system,” i.e., teams, organisations, institutions and their cultures. There are numerous CAS addressing gender equality and diversity and inclusion, with no fewer than 113 schemes across Europe and beyond identified by a recent report (Nason and Sangiuliano, 2020).

Despite this plethora of schemes, there is no agreement nor shared understanding regarding the terminology used. Indeed, there are blurry boundaries between the terms “certification” and “award” schemes. In this paper we opt to define as certification those schemes that assess organisations at multiple points in time, with an element of “renewal,” rather than just at a single point. Single-point assessments are considered to be an “award,” notwithstanding the fact that schemes such as “Total E-Quality award” (in Germany) and the “Athena SWAN” (with the Bronze, Silver and Gold awards) which we perceive as certification, use the word award, thereby adding to any confusion.

In light of the recent announcement from the European Commission where GEPs have become an eligibility criterion for Horizon Europe funding (EC, 2021), there are concerns that their development and subsequent implementation may become a box-ticking exercise. To alleviate this, a wider structure might be needed to ensure that GEPs do not become off-the-shelf products, but are instead implemented and evaluated as drivers for change in organisations and institutions. At national level, discussions are also taking place on the introduction of certification schemes that would help and support institutions in meeting this criterion and advancing gender equality in a more systematic and collective way.

Studies focused on analysing and evaluating gender equality initiatives in higher education have been scarce, and often limited to single case studies, drawing predominantly on Athena SWAN in the UK (Caffrey et al., 2016; Ovseiko et al., 2017; Tzanakou and Pearce, 2019; Ovseiko et al., 2020; Xiao et al., 2020; Drew, 2021). CAS have been underexplored, despite their growing number and potential influence. We know little about how they are operationalised, what their strengths, challenges and impact are to date. Limited efforts have been undertaken to compare and contrast them^1^. This study aims to contribute towards filling this gap by providing a better understanding of the CAS landscape through comparing (gender) equality schemes that target research-performing organisations in two different countries. This paper critically reviews the contribution of CAS in the academy to support gender equity, looking specifically at Athena SWAN and Total E-Quality (TEQ). It enables the identification of the lessons that can be drawn from existing - quite successful in their context - CAS and contributes to the development of comprehensive, impactful CAS that can result in structural and cultural change in organisations.

## Conceptual and Theoretical Considerations

Gender equality initiatives in higher education have proliferated across Europe in the past 2 decades in an effort to address entrenched inequalities in academic systems. However, there is still limited evidence about what works (Bohnet, 2016) and what could lead to meaningful and sustainable structural and cultural change. In this context, GEPs have been recognised as important vehicles for change (Kalpazidou Schmidt et al., 2020) entailing multiple benefits for individuals and organisations:

The process of producing a GEP can provide the setting for reflexivity, consensus-building, and interrogation of the gendered norms (both formal and informal) that underpin the assignment of epistemic authority’ (Clavero and Galligan, 2021, p.)

This said, a comparative critical analysis of GEPs implementation process in different universities have highlighted how context matters in the way GEPs are framed, implemented and enacted (Ní Laoire et al., 2021). Thus, GEPs can be pivotal tools for organisational change insofar as they can be contextualised by reflecting the needs of the local situation of an institution, as well as the wider environment in which these institutions operate. This interaction and co-construction of the micro-, meso- and macro-level has been increasingly highlighted by scholars. Ní Laoire et al. (2021) introduce a conceptual framework demonstrating how macro-level policy and meso-level organisational (gendered) logics can be useful in better understanding how GEPS are interpreted, mediated and (re)produced across different organisational contexts. O’Connor and Irvine (2020) emphasise the significance of gender equality measures simultaneously undertaken at micro-, meso- and macro-level to support change. They alert us to the conditions of “leveraging change”:

“the best possibility of leveraging change arises when measures to promote gender equality are driven at the state (macro); the HEI (meso); and the situational (micro) level simultaneously. Linking state funding to indicators of structural and/or cultural change will help to encourage the use of effective tools to tackle different aspects of gender equality.” (ibid, p16)

The complexity of advancing gender equality involves cultural, structural, institutional and economic factors that create barriers for gender equality in higher education and research - also operating at different levels, micro, meso, macro - to be addressed (Kalpazidou Schmidt and Cacace, 2017). Addressing these barriers requires an equally integrated and sophisticated response–to be operationalised in an evaluation approach that enables a more realistic assessment of the complex ways in which certain gender equality measures promote change. An evaluation is never a value-neutral process, but rather the prolongation of politics by other means. The quality criteria used are not neutral but require political decisions. This adds further to the complexity of certification and to the needs to be considered, by incorporating mechanisms of consensus-building among stakeholders. This is why previous evaluation methodologies and reports of existing schemes (Munir et al., 2014; Graves et al., 2019) have been collected and analysed prior to this analysis, to ensure that it is grounded within the perspectives and recommendations from the wider community of stakeholders.

A certification system does not operate in a vacuum but operates within higher education institutions that are confronted with decreasing public funding which coincides with a heightened need for accountability. The introduction of New Public Management principles that aimed to reduce and streamline a supposedly oversized and inefficient public sector has certainly affected public universities and research institutions (Hood 1991; Newman 2005). A new managerialism tied to the introduction of Total Quality Management principles (Owlia et al., 1997), for example, as well as a marketisation of the public sector have undermined the autonomy and independence of the academy and provoked considerable resistance especially from gender scholars (Thomas and Davies 2002; Anderson 2008; Mountz et al., 2015). The increased resources dedicated to certification can lead to potentially detrimental effects for the advancement of gender equality, where there is resistance and where the exercise becomes devoid of its original value. Ahmed criticises the “new politics of documentation” in this respect, where the circulation of documents related to (race) equality becomes an end in itself and a sign of performance supplanting the actual equality work (Ahmed 2007; Garforth and Kerr 2009; Davis, Kingsbury, and Merry 2010). Equality work can become reduced to paperwork, to “ticking the box” in order to satisfy (external) accountability requirements without engendering real change within the institutions.

The problem goes right to the heart of any certification scheme, in that it needs to be balanced between quality assurance based on process versus quality assurance based on content assessment (Daemen and van der Krogt 2008). Since certification involves standardisation and requires resources, there is always a tendency to focus on easily quantifiable performance indicators instead of context-specific evaluations of content, and the value it represents. In-depth analysis, often requiring costly peer-review, competes with compliance of simple-to-implement indicators that are easy to tick-off but might conceal or even reproduce existing (gendered) power structures (Garforth and Kerr 2009). However, documentation and bureaucratic tasks involved in certification schemes can indeed have a positive impact in terms of contributing to a more transparent organisation, for example in terms of making promotion criteria publicly available (Roth and Sonnert 2011).

It is clear that there can be many benefits to CAS, but it is also crucial to also bear in mind concerns about how CAS might not fulfil the envisaged aim of structural and cultural change in a meaningful and sustainable manner (Ovseiko et al., 2017; Tzanakou, 2019; Tzanakou and Pearce, 2019; Caffrey et al., 2016; Zippel et al., 2016). GEPs, and the wider framework of CAS which promote them, can be gamed and used to reproduce inequalities. This is particularly so when institutions implement activities that just tick boxes and pay lip service while under-represented and marginalised groups are called upon to bear the resulting administrative burden (Tzanakou, 2019; Tzanakou and Pearce, 2019; Ovseiko et al., 2017) sometimes with personal costs for individuals involved in these processes (Tzanakou and Pearce, 2019). This paper will further these debates by exploring CAS as part of macro-level considerations and how they interact with meso- and micro-level considerations comparing two schemes in two different national contexts: the Athena SWAN in the UK and Total E-Quality Award in Germany.

## Setting the Scene: The Athena SWAN and Total E-Quality Schemes

The Athena SWAN (AS) and Total E-Quality Award are both voluntary CAS that are highly esteemed and recognised in their respective national context. Athena SWAN, originating in the UK, is arguably the most prominent and well-known certification system for research organisations, whereas the German Total E-Quality Award extends beyond research with a multi-sectoral base which includes industry and public sector organisations. In this paper we refer to organisations to include HEIs, RPOs but also other organisations that the TEQ targeted.

The Athena SWAN Charter^2^ is a certification scheme that was established in the UK in 2005, aimed at research-performing organisations (RPOs). Its original purpose was to encourage and recognise commitment to advancing the careers of women in science, technology, engineering, maths and medicine (STEMM) employment in higher education and research. In May 2015, the charter itself was expanded to recognise work undertaken in arts, humanities, social sciences, business, and law (AHSSBL), and in professional and support roles, and for trans staff and students. The charter now recognises work undertaken to address gender equality more broadly, and not just barriers to women’s progression. It is applied nationally though has also expanded into Ireland in 2015^3^, the USA in 2017^4^, Australia in 2018^5^ and Canada in 2019^6^ with a pilot in India^7^. A transformed framework was launched for new and existing UK members to use from June 30, 2021. The following description focuses primarily on the post-May 2015 framework, as this was in operation during the period of the CASPER fieldwork and follow-up scenario development and validation process. However, where helpful and relevant, the post-June 2021 version is introduced. According to AdvanceHE, as of July 2021 currently 962 awards in total, with 164 held by institutions and 798 by departments.^8^

Since 2011, there has been a move to link the charter with funding, beginning with the National Institute for Health Research (NIHR) announcing that a Silver Athena SWAN award would be an eligibility criterion for accessing NIHR funding. This requirement has been recently removed to reduce administration during COVID19 pandemic^9^. Although Research Councils UK (RCUK) does not link AS to funding^10^, in January 2013 it launched a statement of expectations that it expects those in receipt of funding to provide evidence of ways in which equality and diversity issues are managed, with recommendations that evidence include participation in schemes such as Athena SWAN and Project Juno.

AS presents three levels of awards, which are available for HEIs and/or their departments. Members are encouraged to work through the levels from Bronze to Silver and Gold. Bronze institution awards recognise that the institution has a solid foundation for eliminating gender biases and developing an inclusive culture that values all staff. Silver institution awards recognise improvement on Bronze level achievement and a significant record of activity and achievement by the institution in promoting gender equality and in addressing challenges across different disciplines. Gold institutions must be beacons of achievement in gender equality and should champion and promote good practice in the wider community. A Gold institutional award recognises a significant and sustained record of activity and achievement addressing challenges across the full range of the institution in and promoting gender equality within and beyond the institution. Both silver and gold applications need to demonstrate AS principles as embedded, with strong leadership promoting and championing the charter principles. Certification is renewable either 3- or 4-yearly, according to the level of the award.

The Athena SWAN Charter is based on ten key principles, and participating institutions commit to progressing the Charter and adopting these principles within their policies, practices, action plans and cultures. A targeted self-assessment framework is used to support applicants in identifying areas for positive action and recognising and sharing good practice. Downloadable resources are provided to enable self-assessment teams in a thorough analysis of their institution’s issues and producing an action plan. The intention is for the framework to empower organisations in identifying the barriers and norms that are unique to their institution and producing targeted actions.

AS Charter award applications are assessed by peer review panels of academics and practitioners that recommend decisions on awards. Both processes and outputs are measured. Evidence for panels includes the themes of communication, senior or high-level commitment, effective analysis of the data, how impact will be measured, self-reflection, honesty, and engagement, based on intersectional qualitative and quantitative data and policy documentation. Consultation is required across the organisation. Clarity of evidence, links to the organisation’s strategic mission and goals and how success was measured and evaluated along with how innovative and sustainable activities are considered. Panels provide unsuccessful candidates with detailed feedback with AdvanceHE operating in a moderating role, supporting internal quality of the process by providing guidance on the application and assessment process and ensuring compliance and consistency. Setting the national legal context is the 2010 UK Equality Act, which includes a Duty aimed at public sector institutions and is associated with reporting requirements and protections for equalities characteristics in addition to protection from discrimination.

The Total E-Quality Award, comprising both an award and a certification scheme, was established in Germany in 1996. It is aimed at the private sector, as well as research and HE sectors. It is presented annually for exemplary human resources management practices that are aimed at providing equal opportunity, with just one level of award. TEQ requires the commitment of organisations to implement equal opportunity-without requiring additional legal guidelines and going beyond already existing guidelines. The award comprises a certificate and an achievement award for sustainability, in combination with the Total E-Quality logo, which can be used by the organisations in all internal and external relations for presentation and image cultivation^11^. Certification also offers a “diversity award”—when applicants address requirements of a newly-introduced diversity add-on module–and an honorary “sustainability award” for organisations that have renewed five times. It aims to measure exemplary activities in terms of human resource management aimed at providing equal opportunity. In this respect, it bears a similarity to the Australian, US and Canadian expansions of Athena SWAN, all of which include diversity dimensions in addition to gender.

In contrast to AS, TEQ is given at a single point in time, on an annual, renewable basis. An award ceremony features a high-profile programme of presentations and discussions and includes a press interview. It is granted for 3 years, with awards thereafter given if a renewed application shows sustainable success and further progress in establishing equal opportunities. In terms of its impact, a survey among award winners demonstrated that the Total E-Quality-Award improves the image of a company and promotes gender equality within the organisation.^12^ As at July 2021, 901 awards have been presented to 339 organisations.^13^

The assessment criteria for TEQ are underpinned by the applicants’ ability to strike a balance between economic requirements and the interests of their employees by implementing suitable human resources strategies to establish equal opportunities A self-assessment tool is provided to give ideas and support, and this sets a series of prescribed questions asking, for example, whether women are employed in scientific and non-scientific managerial positions, are part of selection committees or addressed in tender procedures, or whether women are supported, e.g., in mentoring programmes or through childcare. There are also questions relating to the mainstreaming of gender equality policy into the organisation’s planning and control instruments, such as evaluation procedures and if up-to-date findings from women and gender research are integrated into delivering their research and education. An independent panel of judges then evaluates all applications on behalf of the association and decides on the winners, taking each organisational context into account. Definitions of excellence and quality for the award are based on criteria and standards within the Research-Oriented Standards on Gender Equality developed by the German Research Foundation (DFG, 2008)^14^. These structural and personnel-related standards correspond to the criteria of consistency, transparency, competitiveness and forward-looking orientation, and competence. As a backdrop in Germany there is a national requirement for organisations to implement a GEP which acts as the prevailing legal context and prescribes equality work in a quite detailed manner.

Having provided an overview of the Athena SWAN and TEQ schemes, the paper now outlines the materials and methods it is based upon, before presenting and discussing its findings.

## Materials and Methods

This article draws on the European H2020 CASPER project, which focused on mapping and assessing existing schemes on inequalities (primarily on gender although schemes on various inequalities were explored) and understanding the feasibility of a Europe-wide scheme on gender equality for RPOs. This entailed extensive fieldwork, by a team of interviewers located across four institutions, with qualitative and quantitative data collected from 74 participants during the course of 67 semi-structured interviews, undertaken with key stakeholders and policy-makers throughout the EU and beyond, national machineries for gender equality (e.g. government policy units), research performing organisations and other bodies engaged in existing or past CAS.

For this paper we draw on the overall analysis of the fieldwork and, more specifically, on the 20 interviews dealing with TEQ and Athena SWAN (see Table 1). These included stakeholders running or managing a CA scheme, stakeholders using a scheme (e.g. RPOs, HR, or EDI) and others with involvement in a scheme, policy-makers or known experts on gender issues. Regular fieldwork meetings were scheduled online during the fieldwork to enable partners to discuss potential challenges and coping strategies but also ensure consistency in conducting and analysing the interview data. Consistency in the data analysis and synthesis approach was further supported by the interviewing partners’ experience and expertise in conducting research and qualitative fieldwork on gender issues and diligent adherence to the guidelines. Participants were purposively selected due to their role (e.g. responsible for managing the respective CAS) and/or for their long engagement and experience with respective CAS and gender equality in organisations.

**TABLE 1:** List of interviewees.

Interviewee ID	Certification/Award scheme group	Rule (ie, scheme user, manager, expert)
Interviewee 1	Athena SWAN	User
Interviewee 2	Athena SWAM	Expert
Interviewee 3	TEQ	Manager
Interviewee 4	Athena SWAN	Manager
Interviewee 5	TEQ	User
Interviewee 6	Athena SWAN	User
Interviewee 7	Athena SWAN	User
Interviewee 8	Athena SWAN	User
Interviewee 9	Athena SWAN	User
Interviewee 10	TEQ	User
Interviewee 11	TEQ	User
Interviewee 12	TEQ	User
Interviewee 13	TEQ	User
Interviewee 14	TEQ	User
Interviewee 15	Athena SWAN	User
Interviewee 16	Athena SWAN	Expert
Interviewee 17	Athena SWAN	User
Interviewee 18	Athena SWAN	User
Interviewee 19	Athena SWAN	Manager
Interviewee 20	Athens SWAN	Manager

A topic guide for semi-structured interviews was developed drawing on existing literature, early analysis of CAS and stakeholder identification, with a guide tailored to each of the stakeholder groups. A set of closed questions was developed on key parameters of CAS, creating a set of quantitative indicators on various CAS dimensions. Those who agreed to an interview upon invitation were sent a consent form and participant information sheet by email, and, if requested, the questions they would be asked. Due to the need to “socially distance” because of COVID-19, all interviews were conducted remotely using Zoom or Skype and their built-in recording mechanisms. The study took place between first May and July 31, 2020.

The topic guide included questions regarding 1) the experience and current role of the interviewee (e.g. role, capacity and experience with gender equality certification); 2) their experience, and personal evaluation of the scheme (e.g. strengths, challenges, impact) by the interviewee and 3) their views about the feasibility of a Europe-wide scheme (e.g. whether it is required, its architecture). The procedure for the study was approved by an institutional research ethics committee. Once completed, each interview was written up as a summary or transcript in English and sent to the interviewee for validation and feedback. After agreement was obtained from the interviewee (and any requested changes integrated), the interviewing partners transferred the summary into a de-identified analytic interview summary template. These were collated in an online platform by the authors to review.

For this paper, the authors read the relevant transcripts and summaries and created a coding table providing a critical overview of CAS characteristics (reporting strengths, weaknesses, enablers, challenges, impact, potential for a Europe-wide scheme).

The authors have been involved in CAS as part of gender equality and structural change efforts in their current and past affiliated organisations, which has informed how CAS have been operationalised. While we are critical of the efforts on advancing gender equality in organisations through CAS, which could be seen as moderate feminist practices (Tzanakou and Pearce, 2019), we do consider it important to discuss their strengths, challenges and impact to identify ways to improve them and make them more radical and transformative. In the coding and analysis phases, authors cross-checked the interpretation of participants’ responses to mitigate the infiltration of personal views.

## Results

### The Wider Environment for the CAS

In both the UK and Germany, the wider positive legal environment around gender equality work fostered the engagement of institutions with CAS. In Germany, the national requirement to implement a GEP prescribes equality work in a quite detailed manner. This creates a wider environment where gender equality capacities and resources are already in place. The TEQ is endorsed at a high political level and well-recognised by organisations nationally. The scheme has achieved high levels of adoption and hence there is a positive “critical mass effect” which encourages those who do not have it yet to join. Although TEQ is not required to access funding, the certificate is implicitly “good to have” for evaluations of excellence initiatives. As the scheme has been adopted across research centres, a certain “soft” pressure was described for all institutes to comply. In addition, as the TEQ scheme is well known within Germany, there is scope to generate publicity for the applicant organisation and generate positive public relations. In the UK there is also favorable positive context for sectoral responses such as AS to emerge, particularly since the introduction of the 2010 Equality Act, which is associated with reporting requirements and protections for equalities characteristics. While interest in Athena SWAN was moderate in the beginning, engagement increased steeply when the scheme became linked to access to research funding (NIHR), particularly amidst senior leadership in UK HEIs. Currently, about 15°years later, AS is considered - as one of the interviewees mentioned - as:

“… now embedded enough in UK Higher Education to be significant that people will feel they will have to engage with it, I think it has got to that point now where it is kind of normal and expected.” (Interviewee 1)

Those implementing Athena SWAN cite drivers for involvement as a longstanding shortage of women across STEMM, strong leadership particularly from the HE and research sector and the link to improving practice/best practice around gender equality. It was also felt that the business case is strong, because of potential for gender equality to be linked to better organisational performance.

### Strengths of CAS

Both the TEQ and Athena SWAN schemes present strengths in terms of how they are organised (i.e., the processes and tools involved), and their ability to adjust to the latest developments in gender equality and diversity. Each can be identified as a certification scheme that includes self-assessment and encompasses an intention to improve and advance through progressive approaches and renewals/re-audits, rather than simply assessing achievements in the past. This model was therefore perceived favourably in terms of achieving structural change, in contrast to awards that, due to their time-limited character, do not allow for the follow up and continuous improvement afforded by certification. In terms of process, both schemes require a self-assessment and an audit of the organisation in relation to gender equality. This was perceived as a particular strength since it initiated a reflective process that not only cut across internal committees and processes but also had the potential to engage various stakeholders within the organisation regarding gender equality. An AS user commented in relation to this:

“(The) … Athena SWANprocess has the strength in the process itself. The certification at the end is almost like the icing on the cake, and all of the learning happens in that reflective process looking at the attitude of the people and culture in your department and pulling it apart, and looking at it.” (Interviewee 2)

TEQ users echoed that the scheme was larger than the sum of its parts, with one identifying:

“ … an institutionalisation and reflective process regarding gender equality which transcends the established organisational channels and mechanisms as different working groups are set up.” (Interviewee 3)

Both schemes were also complemented by the comprehensive and structured frameworks they provided for auditing, analysing and designing gender actions plans in both Germany and the UK. The tools used by both the Athena SWAN and TEQ were perceived as particularly flexible as they enabled the tailoring of the application and associated action plans to the specific contextual needs of not only institutional but also departmental levels as highlighted below:

“I do think the department level applications are a really key aspect of Athena SWAN and I do think they drive change because as I said before, so much depends on your local experience and what goes on in that area … ” (Interviewee 1)

“… it’s still context flexible, [Athena SWAN] allows people to progress their work in a way that is appropriate to them while still be recognised by a nationally standardised award” (Interviewee 4)

The structured process and tools used by AS were seen as key to enable teams and institutions starting to work on gender equality because these provide a template and time constraints. As one participant explained:

“ … without a formalised process of analysis they would be re-inventing the wheel but also it could go on forever … ” (Interviewee 2).

AS was providing a place to start, identifying what things those actors responsible for gender change needed to consider, which was seen as important for those without much experience of gender equality work. TEQ was also perceived attractive for “new starters” in equality work since it was felt that the threshold for participation is relatively low, and it was seen as low cost with no need to involve external consultants.

Both schemes were dynamic in terms of reviewing and evaluating their scope and content. TEQ was felt to reflect recent academic developments in gender and equality research, with the diversity add-on being seen as inspiring. One TEQ user commented:

“There is … room to add your own measures which fall outside the specified … areas—such as gender mainstreaming for example. In this sense, it provides a stage to also demonstrate and showcase your commitment to equality beyond box-ticking and standard areas of intervention … ”(Interviewee 5)

Athena SWAN has always been primarily focused on gender and academic staff in STEM, it has expanded its scope to include professional and support staff from all academic disciplines and integrate intersectional analysis where possible and appropriate, at all levels (Bronze, Silver, Gold). The primary focus of AS on gender had served a purpose of drawing attention to entrenched gender inequalities in academia. However, during the CASPER project it was apparent that an evolution to a more intersectional approach was timely, with some participants expressing concerns about a continued, singular focus of attention and resources on gender alone with one stating that though this can be a strength, it can also be detrimental:

“… I am also very aware that by having that focus you are taking resources away from other protected characteristics and that makes me very uncomfortable” (Interviewee 6).

One of the key strengths of Athena SWAN was the requirement to have support from the senior leadership team at departmental or university level since it will affect the application and the approach adopted as illustrated in the following quote:

“The fact that it requires senior buy-in, the first thing you read is that you have a letter from the Head of Department (HoD). That sets the tone for the whole application, the whole process, and I think without insisting on that senior buy-in that again the award would not have the strength that it does and having it linked to funding is the thing that makes people sit up … ” (Interviewee 6).

The TEQ was also felt to offer support to actors that wished to innovate within their respective organisations, with one stakeholder saying that:

“Since the TEQ is not a box-ticking exercise but requires some investment from the organisation, it indeed is perceived as an instrument of real change … external feedback from TEQ in the form of the jury comments as well as through the support given by the interviewee, provides real leverage to get things going and overcome potential resistance from management.” (Interviewee 3)

The AS progressive approach of Bronze, Silver and Gold awards was also seen as a strength for its ability to allow institutions to recognise and demonstrate progress taking in different starting points, which TEQ does not have. Compared to TEQ, Athena SWAN was also favourably commented on for its potential to enable benchmarking and monitoring opportunities (in relation to its structured template) which helped institutions to have a clear focus on the key issues.

### Challenges

TEQ and Athena SWAN shared two main challenges related to assessment/evaluation of applications and support/feedback mechanisms.

TEQ and Athena SWAN users highlighted a lack of transparency in evaluation in relation to criteria, but also in how the evaluation process was consistent across different applications. For example, TEQ implementers considered that the evaluation criteria were not clear in terms of what is applied by the jury to award or reject the certificate. Furthermore, little detail was provided to applicants to justify a rejection and a lack of continuity between subsequent applications. Similarly, AS users reported a misalignment of the AS principles with assessment criteria along with variation of assessment panels that led to inconsistent decisions and feedback, the latter being summarised in the following quote:

“… sometimes some of the feedback that you receive, is just like, you might put it in one application and they (the panel) might say ‘oh that was really good’ and then you might put it in another application and they say ‘why have you done this?’ And it’s like well, but you said it another one that that was a good thing to do, and now in your feedback for another application you are questioning why we are doing this, so I think the consistency needs to be looked at as well” (Interviewee 7)

Linked to evaluation challenges, for both Athena SWAN and the TEQ, concerns were expressed about lack of support and guidance through the process. TEQ users identified limitations on feedback mechanisms from the jury, with assessment reviews being superficial with absence of in-depth feedback to applicants which could help them improve their efforts and actions. There were also limitations in the guidance provided, for example in what activities might be involved and how these could be implemented, with little inspiration for concrete quality work and measures in the organization.

Athena SWAN users also perceived the scheme as not supportive as illustrated by one of the interviewees:

“I think the Athena SWANis very much a ‘pay us the money and we will tick some boxes and we will let you know if you have done alright.’ But you will not get anything from Athena SWAN other than a certificate.” (Interviewee 8)

In relation to the renewal process for both schemes, similar concerns were raised about monitoring the progress between applications. TEQ users highlighted the lack of monitoring the progress of implemented actions or achievement of targets between the first application and the *renewal* application; the application form does not provide room to detail which actions have been achieved and which ones have not. In the Athena SWAN, the previous action plan was not included in the renewal process, but applicants could refer to actions implemented or moved to the new action plan. However, this has changed in the transformed Athena SWAN charter where one of the three sections of the University Renewal Application is devoted to “Evaluation of university’s progress and issues” which asks for reporting progress against previous action plan.^15^

Particular challenges were identified for Athena SWAN in relation to content. There was a need to establish a common set of key indicators (for reporting on or analysing information) and aligning data reporting in ways that avoid creating additional work. While this was sometimes amplified by the need to report intersectional data, the benefits to the Charter from a more intersectional approach were still recognised.

Linked to data collection, the administrative burden of implementing the charter was seen as a challenge, and many voiced concerns about the need to involve more men in gender equality work, as the burden for implementation “*still falls disproportionately to women*” (Interviewee 9), sometimes with a negative effect on their career progression and work life balance in alignment with previous studies (Tzanakou and Pearce, 2019; Ovseiko et al., 2017; Caffrey et al., 2016). In relation to resources and the commitment of staff, it was commented that AS could become more specific about the level of resources committed to AS in terms of budget available for also persons devoted to support application and implementation of AS. Furthermore, users identified difficulty in shifting focus from implementation of the action plan to evaluating actions and understanding impact. There were also concerns about unintended consequences of the charter, including that AS risked becoming a “box-ticking” exercise, and potentially contributing to “gender fatigue” (Kelan, 2009) in RPOs.

### Impact

This reflective process raised awareness of departmental/institutional culture and attitudes and encouraged those involved to take ownership in both schemes. In the case of Athena SWAN, it was also widely perceived as having raised awareness of gender equality issues more broadly across in the higher education sector, allowing people to feel comfortable about discussing gender matters:

“I think you would be hard pushed to find someone within our department who has not heard of Athena SWANand hasn’t heard of gender equality, so I think as I say it has really brought it to the forefront … ” (Interviewee 6)

AS users interviewed identified a wide range of impact in their institutions, including increased representation of women in senior positions and decision-making bodies–in alignment with previous research (Gregory-Smith, 2018) -, more gender-balanced shortlisting and appointments and greater investment in training for women, which improved their promotion prospects, with one interviewee confirming:

“We invest more in our training; we send women on courses like women transforming leadership. We have sent 11 women on that course and every single one has now been promoted, had significant promotion, has won significant grants since being on those courses.” (Interviewee 6)

A TEQ user identified that the certification could provide a competitive advantage, and for example “… *might be attractive for attracting talent as they show that the organization is committed to equality.*” (Interviewee 5).

Respondents with experience of TEQ and AS also recognised the challenge of attributing change and impact to a specific CAS when other equality work is taking place, where organisations might already have a strong equality culture. For example, a representative of an organisation that had increased the percentage of women in leadership positions considerably noted positive developments, but were unable to demonstrate an actual causal relationship with the implementation, as “these can’t really be tracked back to the certificate (TEQ) only” (Interviewee 5).

In the case of Athena SWAN, some observed that the wider community did not recognise its impact because of a lack of “branding.” One interviewee, for example, recollected the following:

“So, for example in one department which is coming up to renew their silver award, obviously for silver you have to look for impact and they were talking to people and trying to see ‘what do you think Athena SWAN has done?’ They had one comment from I think a senior academic basically saying that ‘Athena SWAN is a waste of time, I know you guys are on that committee, but I have not seen you do anything as far as I know you just write this application, you get an award, it doesn’t seem to mean anything.” So, they then said, ‘well what about all these activities and bring your children to work day or the support for postdocs going to conferences in terms of travel funding or x, y and z’. Then the person said, ‘I think all those are brilliant and great, I just did not realise that they were from Athena SWAN’. So … and sometimes I think there is an issue around….there is a discussion to be held around branding….” (Interviewee 1).

## Discussion

O’Connor and Irvine’s work (2020) identifies the necessity of drivers at macro-, meso- and micro-levels to successfully leverage change. The success of both TEQ and Athena SWAN can be viewed within this framework. For the TEQ, endorsement at a political level and the national requirement to implement a GEP creates an environment where gender equality is a societal and business goal. In the case of Athena SWAN, a sectoral top-down response by higher education institutions in the UK was supported by the wider ecosystem when the 2010 Equality Act created expectations for proactive equalities work. While AS was slow at getting traction in the first few years after its creation in 2005, the link established in 2011 with research funding led to all UK HEIs developing an active interest in securing an award. This fostered the development of associated organisational networks and stakeholder buy-in, with structures that support the development of gender expertise. At the meso-level, organisations themselves are motivated to implement the scheme by seeking to gain a competitive advantage, for example through enhanced reputation and attracting staff. Creating this favourable environment can become a particularly desirable outcome for institutions in an increasingly marketised sector. A wider societal and organisational ethos of gender equality as the norm will, in turn, support attitudinal change in individuals at the micro-level, and empower these organisations to challenge inequalities.

A key differentiation between AS and TEQ was that the former was a sector-specific response to challenges in higher education, while TEQ targeted organisations beyond HEIs and RPOs. This had implications for the drivers and the framing as to why organisations should engage with these CAS. It was notable that the TEQ users identified the reputational and talent acquisition aspects of the award, therefore focusing on more generalised “business case” benefits, which may reflect the multi-sectoral nature of TEQ and its implementation across a non-homogeneous group of organisations. On the other hand, some drivers that were apparent for Athena SWAN, such as a shortage of women in STEMM, were not identifiable in feedback from the TEQ stakeholders (consisting of research and public administration institutions). That this was not an issue to them may be attributable to the pre-existence of a strong culture of gender equality supported by the widespread GEP implementation.

Gender equality work is dynamic and co-constructed between the macro- and meso-level as demonstrated in the two CAS compared herein. When AS was first introduced, the Bronze award (the first award in AS) was considered as having a low threshold - as with the TEQ - with the aim of recognising applicants’ efforts to start collecting, analysing and reflecting on data, identifying challenges and developing the actions relevant to their institution. However, the expansion and success of institutions and departments in securing AS has led to concerns about “shifting goalposts,” with a much higher threshold in terms of requirements in achieving a Bronze award (Pearce, 2017). The dissemination and exchange of knowledge on gender equality work and actions and their subsequent adoption and transferability in different institutional contexts led to raising the bar even higher, beyond that which had been required in the past. While this was unfortunate for departments and institutions that felt let down by this process, it is important to welcome a shift in standards that contributes further to gender equality. What is important for retaining engagement and commitment, though, is to remain transparent and clear about such a shift.

In the light of the recent EC announcement that GEPs will be required for accessing Horizon Europe funding from 2022 and the possibility that these GEPs might be linked with and/or supported by a Europe-wide CAS - to mitigate any risks of off-the-shelf GEPs and box-ticking exercises - what can we learn from existing schemes such as TEQ and AS? Discussions during the CASPER fieldwork indicated that, at national level, the introduction of certification schemes to support institutions in meeting GEP criteria and advancing gender equality in a more systematic and collective way are already taking place. It is therefore helpful to identify several important learning points which could feed into the development of a Europe-wide CAS scheme.

A willingness to remain dynamic and able to evolve is key. This has been demonstrated in multiple ways through the AS and TEQ. It has been clearly illustrated by activity since the completion of the CASPER fieldwork in July 2020, since both TEQ and Athena SWAN have been working on updating their content. In June 2021, the Transformed Athena SWAN Charter was introduced with a series of questions on intersectionality being included in applications (at all levels) while in TEQ, the PDF application required is currently converted into an online portal that will not only focus on gender but will capture diversity issues as well. The Transformed Charter has relied on recommendations from a wide sector consultation and various evaluation exercises by external organisations that Advance HE procured to understand the impact of the scheme in the sector and identify ways for further improvement^16^. Stakeholders driving CAS should continuously reflect and review on whether the CAS are relevant, appropriate and responsive - e.g., continue to seek feedback from users - to the dynamic needs of organisations in relation to advancing gender equality.

A comprehensive framework with structured processes and tools was pivotal in encouraging even new starters in gender equality work to make their first steps. Flexibility and allowing for the tailoring of actions to the local context was seen as key for CAS to achieve change taking the specific needs and challenges into account, as highlighted by Ní Laoire et al. (2021). Thus, policy-makers and stakeholder organisations must ensure a balance between standardisation (structured templates, tools, processes) and flexibility to contextualise gender equality actions. This would need to be a primary consideration in the diverse landscape of organisations in the European higher education and research area for a Europe-wide scheme to be appealing and have potential to bring about change (Tzanakou et al., 2020).

Support and guidance through the certification process was seen as pivotal to help organisations to improve and learn from their efforts rather than simply focusing on how to get the award. Tailored feedback and advice to organisations was welcomed at all stages of certification, from preparing the application (e.g., how to collect and analyse data) and the design of GEP to the implementation and evaluation of activities. This support could be provided through various means such as: individuals with experience in gender equality work acting as critical friends across organisations and within communities of practice, online help desks, site visits with peers/experts (see for example Project Juno^17^), a library of actions for inspiration can all contribute to developing communities of organisations that reflect, help and learn from each other, especially for organisations with limited resources. For a Europe-wide CAS, this could take the form of potentially introducing a responsive and accessible helpdesk (national and/or EU contact points) to support and address queries and doubts, building on expanding and developing further existing communities of practice, gender experts^18^ and EU-funded projects on structural change^19^.

The resource-intensive character of gender equality work has been a key challenge for organisations that need to self-assess, design, implement and evaluate a GEP (Tzanakou and Pearce, 2019). Requirements for increased documentation and data collection can become burdensome (Ahmed, 2007), but equally, the impact identified by participants in the CASPER fieldwork shows that this work can lead to more than a box-ticking exercise. As this paper shows, resources are significant within the bodies that own and coordinate CAS as well. Greater resources should be allocated or redirected within the CAS to address two key challenges of applying organisations, which are: 1) resources for more feedback, tailored advice and guidance to the design, implementation and evaluation of organisational GEPs and 2) resources to ensure that evaluators of applications are appropriately trained and have transparent evaluation criteria and processes to ensure consistency and trust towards the scheme.

While interviewees identified various impacts of CAS in relation to: raising awareness on gender equality and intersectionality, stimulating discussions around the topic, increasing representation of women in senior posts and training opportunities for women, and becoming more “family-friendly,” enhancing the attractiveness of the organisation, there were many challenges in attributing impact of AS and TEQ to the scheme alone. Amongst the reasons for this challenge was that activities were not always branded under the scheme, particularly in the case of Athena SWAN. What should be noted is that the “branding” of TEQ and Athena SWAN was very successful at macro level, at national and for Athena SWAN at international level considering the adoption of the scheme in Ireland, the USA, Canada, Australia and the pilot in India. However, within institutions (meso-level) and at the micro-level, the branding seemed to be less utilised and thus less effective, increasing the challenge in identifying the benefits of the schemes compared to other initiatives and activities.

Identifying causality between actions and outcomes/impact in the context of gender equality and organisational change will remain challenging due to the complex nature of these activities. Future CAS and efforts should therefore be underpinned with logic frameworks, multiple theories of change and, more importantly, with combined expertise in the fields of organisational change, psycho-social theories, network theories, programme evaluation and many more (Laursen and Austin, 2020; Kezar, 2018).

This paper has contributed towards a better understanding of the CAS landscape, and is the first to compare CAS relating to gender and the development of comprehensive, impactful CAS that can result in structural and cultural change in organisations.

In summary, we argue that CAS should consider several key factors. Firstly, gender equality work is dynamic and co-constructed between the wider environment and the institutions responsible for its implementation and must continuously evolve. It is pivotal that CAS have flexibility in content that allows local contextualisation. Support and guidance for those implementing CAS is essential, through providing resources, training and inspiration. Those driving a CAS need to reflect regularly on the relevance and responsiveness of their schemes, preferably through consultation and evaluation. Maintaining clarity and staying transparent as these processes change are essential to keep users engaged and committed. Gender equality is a complex phenomenon to investigate, thus it requires a range of expertise coupled with comprehensive frameworks informed by theories of change to make the benefits of implementation more easily recognisable and thus enhance the confidence of users.

Limitations. The data collection comes from within a framework of RPOs, where TEQ organisations, though research-performing, are operating in a broader, business-based system when implementing the multi-sectoral TEQ award/certification. Nevertheless, the review of these schemes and the feedback of their users, owners provide a useful framework through which to identify learning for future CAS on gender equality.

Whilst our sample is limited, this was based on the a-priori selection of participants whose role, expertise and experience with CAS enabled them to provide rich accounts from different perspectives (managing the CAS, applying to the CAS, etc). This provided expert insights into the operationalisation and understanding of CAS, which is the main purpose of this paper.

## Data Availability

The datasets presented in this article are not readily available to avoid identification of interviewees, Requests to access the datasets should be directed to ctzanakou@brookes.ac.uk.

## Footnotes

1 One previous study (Rosser et al., 2019) makes a comparison and looked at the effectiveness of and lessons learned from Athena SWAN and ADVANCE, but the latter is a grant rather than certification and award scheme

2
https://www.ecu.ac.uk/equality-charters/athena-swan/

3 See Drew, (2021). Navigating unchartered waters: anchoring Athena SWAN into Irish HEIs. Journal of Gender Studies, DOI: 10.1080/09589236.2021.1923463

4 The SEA Change initiative, inspired by Athena SWAN and piloted in 2017 https://seachange.aaas.org/

5
https://www.advance-he.ac.uk/news-and-views/SAGE-athena-SWAN-gender-equity

6 Dimension, https://www.nserc-crsng.gc.ca/NSERC-CRSNG/EDI-EDI/Dimensions-Charter_Dimensions-Charte_eng.asp

7
https://www.natureasia.com/en/nindia/article/10.1038/nindia.2020.41

8 AdvanceHE (2021). Athena Swan Charter participants and award holders, https://www.advance-he.ac.uk/equality-charters/athena-swan-charter/participants-and-award-holders#:∼:text=There%20are%20currently%20962%20total,and%20798%20held%20by%20departments

9 More can be found here: https://www.nihr.ac.uk/news/nihr-responds-to-the-governments-call-for-further-reduction-in-bureaucracy-with-new-measures/25633

10 Athena SWAN is linked to funding in Ireland far more comprehensively than in the UK: https://www.sfi.ie/research-news/news/irish-funding-bodies-to-require-athena-swan-gender-equality-accreditation-for-higher-education-institutions/

11
https://www.total-e-quality.de/en/award/award/

12 Technical University Dortmund, https://www.total-e-quality.de/media/uploads/global_assets/teq_10punkte.jpg

13
https://www.total-e-quality.de/en/award/award/#:∼:text=Organisations%20from%20the%20private%20sector,been%20presented%20to%20339%20organisations

14
https://www.dfg.de/download/pdf/foerderung/grundlagen_dfg_foerderung/chancengleichheit/forschungsorientierte_gleichstellungsstandards_2017_en.pdf

15 More information can be found at the Transformed UK Athena SWAN Charter: Information pack for universities, available at: https://s3.eu-west-2.amazonaws.com/assets.creode.advancehe-document-manager/documents/advance-he/AdvHE_AS%20University%20info%20pack_FINAL_1625130696.pdf

16 For example, an independent evaluation into the impact and effectiveness of the Charter by Loughborough University in 2014 https://www.ecu.ac.uk/wp-content/uploads/external/evaluating-the-effectiveness-and-impact-of-the-athena-swan-charter.pdf; and In 2018, Ortus Economic Research and Loughborough University https://www.advance-he.ac.uk/knowledge-hub/impact-evaluation-athena-swan-charter-2019, the latter concluding that there was a need to streamline the application process, reduce the administrative process and increase both the consistency and transparency of the award and levels of support

17
https://www.iop.org/about/IOP-diversity-inclusion/project-juno#gref

18 For example, Gender Academy and ACT communities of practice

19 Including PLOTINA and GEARING-Roles
